# Spatial Variation in Agricultural BMPs and Relationships with Nutrient Yields Across New York State Watersheds

**DOI:** 10.1007/s00267-024-02008-x

**Published:** 2024-07-02

**Authors:** Rebecca Schewe, Lidiia Iavorivska, Christa Kelleher

**Affiliations:** 1 Syracuse University, Syracuse, NY USA; 2 Pennsylvania State University, University Park, TX USA; 3 Lafayette College, Easton, PA USA

**Keywords:** Spatial, Water quality, Agriculture, BMPs, NY

## Abstract

Agricultural nutrients nitrogen and phosphorus can subsequently be transported to waterways and are often managed through the adoption of best management practices (BMPs). However, we have a poor understanding of how the use of BMPs varies spatially and how BMP adoption might be related to nutrient yields in surface waters. To address this, we performed a survey of agricultural landowners across New York State and compared this with estimates of annual incremental nitrogen and phosphorus yields of agricultural origin from the Spatially Referenced Regressions On Watershed attributes (SPARROW) model. Using these socio-behavioral data and SPARROW predictions, we perform colocation analysis to identify areas where watersheds with high nutrient yield from agriculture are collocated with non-use of agricultural BMPs. This colocation analysis offers a novel methodology for identifying areas where monitoring of waterways and promotion of best management practices could be targeted to achieve the greatest benefits.

## Introduction

Agriculture, including practices such as the application of animal manure and synthetic fertilizers, is a primary cause of nitrogen (N) and phosphorous (P) pollution in waterways (Carpenter et al. 1998; Laitos and Ruckriegle 2012; Longhurst 2012; Sharpley and Jarvie 2012). While N and P are essential nutrients for crop growth, excess N and P are transported to groundwater and surface water, where high amounts of these nutrients can lead to negative environmental consequences (Chislock et al. 2013). Excess N and P can stimulate the growth of algae, depleting water oxygen levels and having deleterious impacts on aquatic community composition (Smith et al. 1999; Dodds and Smith 2016). Due to toxin production by algae, large algal blooms can also threaten human health (Michalak et al. 2013). These environmental consequences of eutrophication combined with water body degradation cost the US billions of dollars in annual economic damages (Dodds et al. 2009). Excessive inputs of bioavailable forms of N and P also have far-reaching effects on biodiversity and terrestrial and aquatic ecosystems) through altered environmental stoichiometry that impacts food chains), population dynamics, and even organism genomics (Guignard et al. 2017).

Despite the recognized impact of agricultural practices on nutrient enrichment in waterways, agriculture in the United States (US) is largely exempted from water-quality regulations and standards and classified as a “non-point” source of pollution (Longhurst 2012). In New York State (NYS), the domain of this study, the Department of Environment and Conservation (DEC) reported that non-point sources, including agriculture, were responsible for 90% of the water quality impacts on rivers and streams in 2008 (Centner 2012, p. 326). In this study, we examine a suite of agricultural conservation practices and nutrient yields in watersheds of NYS to identify spatial patterns of conservation behaviors and water nutrient yields. Using spatial analysis and a dataset that combines social and environmental measures, we offer a novel methodology for identifying areas of concern for future monitoring and/or targeted intervention concerning agricultural conservation behaviors to reduce nutrient yield in watersheds. This spatial analysis offers a novel approach for identifying priority areas for monitoring and/or targeted intervention by identifying areas in which high nutrient yields co-occur with low adoption of conservation behaviors. State agencies and conservation partners have limited resources and targeting interventions in areas with the highest potential impact can maximize the effectiveness of campaigns to improve water quality outcomes.

The overall impact of agriculture and conservation behaviors on water quality is clearly established (Carpenter et al. 1998; Lovell and Sullivan 2006; Laitos and Ruckriegle 2012; Sharpley and Jarvie 2012; Logan et al. 2018; Zak et al. 2018), but limited studies have directly examined how agricultural conservation behaviors may vary spatially and how that may relate to nutrient yields in waterways. In one such spatial study, Gurdak and Qi (2012) find that recently recharged groundwater is vulnerable to nitrate contamination from agricultural sources, with the relative impact of agricultural practices on groundwater shaped by biogeophysical characteristics (Gurdak and Qi 2012). Other work has shown spatial variation in the impact of conservation tillage – one of the focal BMPs in this study – on water quality, further suggesting the importance of local biogeophysical characteristics to modulate or augment downstream pollution (Yang et al. 2005). Zimmerman and colleagues (2019a) demonstrate the potential efficacy of spatially targeted BMPs in the US Cornbelt region, as well. At the scale of the continental US, work by Cheng et al. (2020) estimated that increasing wetland coverage in spatially explicit areas has the potential to accelerate N removal. We extend this literature by examining spatial patterns of BMPs and nutrient yields in NYS, a region with extremely high density of surface waters (New York State Department of Environmental Conservation 2024). Importantly, we do not attempt to offer a causal analysis linking specific agricultural BMPs to specific nutrient yield outcomes, but rather to develop a spatial toolset that could be used to identify priority areas for future monitoring or interventions: areas with both low adoption of BMPs and high levels of N and P yields from agriculture.

In this study, we examine the spatial variation in three BMPs and nutrient yields in NYS using a survey that is representative of farmers across NYS and nutrient yield estimates generated by SPARROW models (Schwarz et al. 2006). Specifically, we aim to characterize the utilization of three widely-used BMPs: 1) the use of conservation tillage and no-till methods (Yang et al. 2005; Logan et al. 2018; Kelly et al. 2019; Cooper et al. 2020); 2) the use of regular soil testing for nutrient content (Gartley and Sims 1994a; Sims 1998a; Maguire and Sims 2002); and 3) the use of buffer zones or conservation cover around waterways (Norris 1993; Lovell and Sullivan 2006). We examine agriculturally derived incremental N and P yields (which are input loading of nutrients to a stream from the surrounding landscape, normalized to watershed area) across 1411 NYS subwatersheds using the SPAtially Referenced Regression On Watershed attributes (SPARROW) model (Schwarz et al. 2006). Specifically, we hypothesize that (1) areas with low usage of BMPs will be collocated with areas having high levels of N and P in waterways, but that (2) the relationship between BMPs and N and P yields in waterways will vary spatially, reflecting local biogeophysical conditions.

## Study Area

This study is focused within NYS, a state which features abundant water resources, with rivers draining to the Great Lakes, the Ohio, Delaware, and Susquehanna River basins, and the Atlantic Ocean. NYS has a water area of 19,240 km^2^, or 13.6% of the state’s total surface area (United States Census Bureau 2024), with over 7600 lakes and reservoirs, including portions of two Great Lakes, and more than 110,00 km of rivers and streams (New York State Department of Environmental Conservation 2024). Approximately 16% of NYS land area is used for agriculture, roughly evenly split between hay or pasture and cultivated crops (see Supplementary Information for figure) (Multi-Resolution Land Characteristics Consortium 2019; United State Geological Survey 2021). Our findings in this water-dense and geomorphologically diverse state can inform BMP promotion in other parts of the Eastern US and elsewhere where agricultural production coexists in proximity to a large concentration of surface waters.

## Selection of BMPs

A wide range of conservation behaviors and BMPs have been demonstrated to affect nutrient transport from agricultural fields to watersheds, in this study we examine just three, selected for both their demonstrated relationship with nutrient levels in watersheds and their socio-political importance. The first BMP we examine is the use of conservation tillage and/or no-till methods. The primary goal of conservation tillage, including no-till, is to reduce soil erosion (Gebhardt et al. 1985), but decreased runoff is also a significant benefit of conservation tillage (Kukal et al. 1991; Duiker and Myers 2005; Logan et al. 2018; Cooper et al. 2020). Reduced tillage has been consistently shown to reduce runoff and nutrient transport to waterways (Wendt and Burwell 1985; Choi et al. 2003; Raczkowski et al. 2009; Williams et al. 2009; Busari et al. 2015; Sun et al. 2015; Carretta et al. 2021). Conservation tillage and no-till methods are also heavily promoted by both federal conservation programs (Crowell 2020; No-Till Farmer 2023) and state extension services. The second BMP we examine is the use of soil testing for nutrient content. Regular soil testing provides the foundation for nutrient management more broadly as it determines the existing levels of nutrients in agricultural soils (Peck 1990; Havlin 2020). Soil testing has been found to be particularly strongly linked with decreased P loss from soil to water (Gartley and Sims 1994b; Sims 1998b) but has also been shown to effectively reduce nitrogen leaching to waters (Power et al. 2000; Lord et al. 2002). Soil testing is also heavily promoted by both federal and state conservation programs (Natural Resources Conservation Service 2009). Our final focal BMP is the use of planted buffers around surface waters. The use of planted buffer zones has been consistently shown to reduce the transport of both N and P into surface waters (Norris 1993; Lovell and Sullivan 2006; Mayer et al. 2007; Walton et al. 2020). Buffer zones have been shown to be effective across a wide range of soil types and conditions and are again heavily promoted as part of federal and state agricultural conservation programs (Natural Resources Conservation Service 2023). These three BMPs coexist with a number of other conservation behaviors and biogeophysical conditions that may also affect N and P yields from agriculture. In our analysis, we examine each BMP in isolation to test a novel methodology for identifying areas of concern for both BMP usage and nutrient yields, but future research should further examine conservation practices in concert and further specify their relationship with biogeophysical conditions.

## Materials and Methods

### Survey Data: BMP measures and controls

The adoption of BMPs and other control variables are measured via self-reported responses to a mail survey. With the services of the Survey Research Institute (SRI) at Cornell University, between February and April 2019 the Biosolids and Manure survey was sent to a stratified random sample of NY agricultural landowners (see Supplementary Information), using a contact list purchased from Farm Market ID that included both commodity produced and farm size. Farm Market ID is an agricultural marketing company that collects and sells data about farm operations. Survey research received ethics approval from the Syracuse University Institutional Review Board and participants received informed consent to participate and publish.

To ensure a representative sample with adequate variation in farm size and commodity, the sample was stratified to include the four largest commodity systems in NY and further stratified by size (see Supplementary Information) (USDA National Agricultural Statistics Service 2017). A total of 1474 usable surveys were returned, for an AAPOR response rate of 4 of 27.1% (American Association for Public Opinion Research 2016). Non-response analysis revealed no significant patterns in non-response by key characteristics of farm size and commodity and the resulting respondents were found to be representative of NYS farmers.

The survey included questions concerning: (1) farm characteristics, (2) the use of BMPs, (3) manure and biosolids management, (4) water quality concerns, and (5) sociodemographics. The survey took approximately 15 min to complete and was sent using a modified Dillman (Dillman 2007) Tailored Design Method including a full-color cover, first-class postage, a stamped return envelope, and a $2 bill incentive in the first mailing, and five follow up mailings. Each survey response was geolocated to the farm address. This farm address may not represent the specific field location in which BMPs were applied.

### Spatial analysis of survey respondents and BMPs

Examining spatial patterns amongst respondents, we used the Nearest Neighbor Index in ArcMap 10.7 (ESRI 2022a) to assess the overall spatial clustering of respondents. A Nearest Neighbor Index of less than 1 indicates that there is spatial clustering of respondents in which respondents are more similar to their neighbors on the measures in the data set than if they were spatially random. The Nearest Neighbor Index is a global measure, meaning that it assesses the spatial clustering of respondents using the entire dataset.

### Nutrient yields data

We extracted measures of nutrient levels in waterways as incremental yields (kg km^−2^ yr^−1^), defined as area-adjusted yields of total N and total P delivered to the streams from agricultural sources located in the immediate watershed area. These values were derived from USGS SPARROW models for Northeast and Midwest regions covering portions of the NYS (Ator 2019; Robertson and Saad 2019). SPARROW uses a range of spatially distributed data on biogeophysical conditions of watersheds, pollution sources, and land management as explanatory variables in nonlinear regression equations to predict the fluxes of water, nutrients, and sediment from the landscape into streams and their modification as they move through the river network (Schwarz et al. 2006). The incremental yields generated by SPARROW include loads from the upgradient land draining to each reach and is therefore a measure of yields resulting from local pollution sources excluding upstream sources (USGS 2019). To remove the impact of watershed size on yields, we normalized each yield to the watershed area to produce yields (units of kg km^−2^ yr^−1^).

SPARROW has been demonstrated to provide valid estimates of streamflow, total and incremental N, total and incremental P, and sediment across the regions of conterminous U.S. (Preston et al. 2011; Saad et al. 2011). While the inputs for the SPARROW model setup were focused on the year 2012 (representing conditions for an “average” year), the model-predicted nutrient fluxes were detrended to be representative of long-term average annual hydrologic and climatic conditions across the model domain (Ator 2019; Robertson and Saad 2019). To generate point data for SPARROW outputs, for this study, SPARROW data was assigned to the HUC12-level watershed centroid.

### Spatial clustering of SPARROW outputs

To examine the spatial clustering of SPARROW outputs, we estimated Anselin Local Moran’s I in ArcMap 10.7 (ESRI 2022b). Local Moran’s I identifies statistically significant spatial clusters of point data (HIGH-HIGH and LOW-LOW) and outliers (HIGH-LOW and LOW-HIGH) for continuous measures. We report clusters and outliers at the 95% significance level and above.

### Delineating Areas of High Nutrient Yields

To operationalize watersheds with high yields of N and P from agriculture, we assigned watersheds a value of 1 if the incremental nutrient yield from agriculture (based on SPARROW output) is more than 1 standard deviation above the state mean for that nutrient. Otherwise, the watershed was assigned a 0.

### Colocation of High Nutrient Yields and Lacking BMPs

To examine the spatial relationship between agricultural BMPs and incremental N and P yields from agriculture, we utilized colocation analysis in Arc GIS Pro 3.0 (ESRI 2023). Colocation analysis is designed to measure the extent of colocation (or isolation) of two categories of point data. Colocation analysis generates two types of colocation quotients: a global colocation quotient (GCLQ) and a local colocation quotient (LCLQ). Each is discussed in greater detail below. Importantly, these colocation quotients only address the colocation of BMP presence or absence for survey respondents and do not include agricultural production that is not represented in the survey data.

#### Global Colocation Quotient

A GCLQ is a measure of the “overall colocation pattern between any two types of point objects” (Hu et al. 2018, p. 433) to determine if the two categories occur as nearest neighbors to each other more frequently than by chance. In other words, GCLQ looks at a specific category of point data and a second specific category of point data to determine if the two categories are collocated more than if the relationship was spatially random. A GCLQ that is greater than 1 indicates a pattern of overall colocation that is greater than expected to randomly occur and a GCLQ of less than 1 indicates a pattern of isolation that is greater than expected to randomly occur (Hu et al. 2018). In our analysis, we calculate GCLQ to measure the extent of colocation between the absence of an agricultural BMP (a survey respondent who is NOT using the BMP) and a watershed with an N or P yield that is more than 1 standard deviation above the state average for that nutrient.

#### Local Colocation Quotient

A GCLQ represents the overall spatial colocation patterns, but those colocation patterns may themselves vary across space. An LCLQ is also a measure of colocation between two categories of point data, but an LCLQ is calculated for each specific point and therefore can vary across space. Thus, an LCLQ can reveal spatial variation in colocation patterns, enabling us to identify whether there are stronger relationships between high N and P yields and BMP adoption in certain parts of NYS. Similar to the GCLQ, an LCLQ of greater than 1 indicates colocation between the two categories of point data and an LCLQ of less than 1 indicates isolation (Hu et al. 2018). In our analysis, we report the distribution of LCLQs for each BMP and an N yield of more than 1 standard deviation above the state mean, a P yield of more than 1 standard deviation above the state mean, and the statistical significance of the LCLQs.

## Results

### Survey outcomes

Non-response analysis found no statistically significant differences between survey respondents and non-respondents in farm size or commodity. To confirm the representativeness of survey respondents, we also compared respondents to the 2022 Agricultural Census for NY state and found no significant differences in farm size (see Supplementary Information). Survey respondents included 321 hay farms, 317 beef farms, 403 dairy farms, and 422 corn farms. A total of 58 of NY’s 62 counties were represented amongst respondents.

As expected in NY state, only 23% of respondents reported that their property does not border surface water, and 1% reported that they were unsure. The most commonly reported surface water was a river/creek (44% of respondents). Of those respondents who report that their property includes or borders surface water, 50% report that they use unplanted buffer zones around surface water, and 20% report that they use planted buffer zones.

Across respondents, the most commonly reported soil types are clay (30%), clay-loam (28%), and loam (16%). A minority (23% fall, 31% spring) of respondents practice no-till methods, while moldboard (19% fall and 29% spring) and disk (11% fall and 32% spring) are the most common non-conservation types of tillage. Overall, 62% of respondents use some form of conservation tillage (no-till, ridge-till, or mulch till). A minority of respondents (24%) report never testing soil for nutrient content, 39% report testing soil every 1 to 5 years, and 26% less often than every 5 years. Only 5% reported testing soil every year.

Average Nearest Neighbor analysis reveals significant spatial clustering of survey respondents. The Nearest Neighbor Index is 0.58 (*p* < 0.001), indicating that respondents are significantly more similar to their neighbors than if they were spatially random. Survey cases cover a total of 757 HUC12 watersheds across NY state, with an average of 2 farms per watershed, a maximum of 10 farms in 3 watersheds, and 376 watersheds having only 1 farm. Survey respondents had an average of 186 planted acres per HUC12 watershed, with a maximum of 4586 planted acres in one watershed and twenty watersheds having fewer than 5 planted acres from survey respondents.

### SPARROW estimates of N and P yields in waterways

While the SPARROW model and associated output are publicly available (Ator 2019; Robertson and Saad 2019), we briefly summarize the outputs of this model for NYS (see Supplementary Information). Approximately 11.5% of all HUC 12 watersheds had zero contributions of N from agricultural sources, while 22.0% of all watersheds had more than 50% of total N estimated to be supplied from agricultural sources. The average incremental N from agriculture was 660 kg/km^2^ with a standard deviation of 567 kg/km^2^. Watersheds with an incremental N from agriculture of greater than or equal to 1,228 kg/km^2^, therefore, meet our threshold of concern. Contributions of N from agriculture were generally the largest in western and central NY. Approximately 12.8% of all HUC 12 watersheds derived no P from agriculture, while 17.4% of watersheds derived more than 50% of total P from agricultural sources. The average incremental P from agriculture was 23 kg/km^2^ with a standard deviation of 56 kg/km^2^. Watersheds with an incremental N from agriculture of greater than or equal to 79 kg/km^2^, therefore, meet our threshold of concern. Watersheds located in western NY tended to have a higher fraction of P sourced from agriculture.

**Fig. 1:**
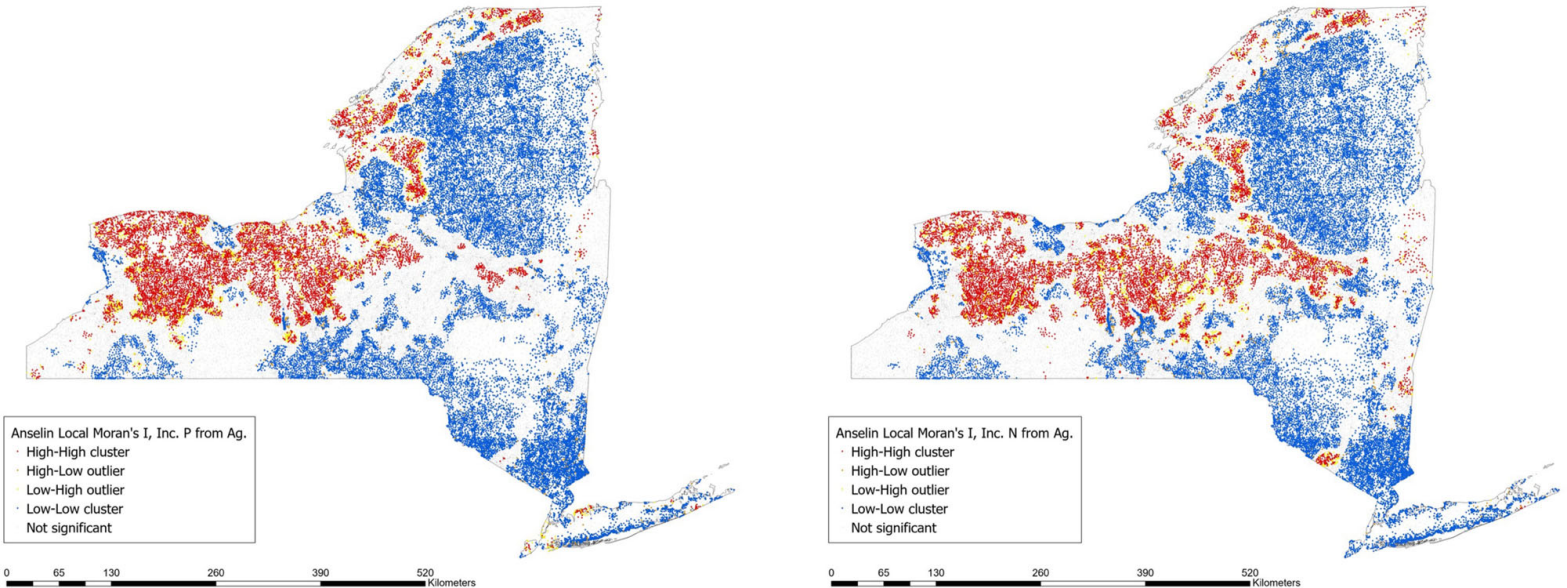
Anselin Local Moran’s I of incremental N and P yield from agricultural sources

Anselin Local Moran’s I results indicate significant spatial clustering of both incremental N and incremental P from agricultural sources in NY HUC12 watersheds. Examining incremental N from agriculture (Fig. 1), Anselin Local Moran’s I results show that 18% of HUC12 watersheds (8925) are located in HIGH-HIGH clusters (shown in red) and 50% of HUC12 watersheds (24,921) are located in LOW-LOW clusters (shown in blue). Only 1% of HUC12 watersheds (491) are located in HIGH-LOW clusters (shown in orange) and 4% of HUC12 watersheds (1893) are located in LOW-HIGH clusters (shown in yellow). Finally, 28% of HUC12 watersheds (14,073) have no significant spatial correlation with neighbors regarding incremental N from agriculture.

Examining P yields from agriculture (Fig. 1), Anselin Local Moran’s I results show that 18% of HUC12 watersheds (9115) are located in HIGH-HIGH clusters (shown in red) and 49% of HUC12 watersheds (24,443) are located in LOW-LOW clusters (shown in blue). Only 1% of HUC12 watersheds (518) are located in HIGH-LOW clusters (shown in orange) and 4% of HUC12 watersheds (1959) are located in LOW-HIGH clusters (shown in yellow). In addition, 28% of HUC12 watersheds (14,268) are located in areas with no significant spatial correlation with neighbors regarding incremental N from agriculture. In other words, SPARROW results have high spatial autocorrelation for estimates of incremental N and P yields from agriculture, but there are outliers located throughout the state.

### Colocation of High Nutrient Yields and BMPs: Nitrogen

#### Use of No-Till Methods

**Fig. 2:**
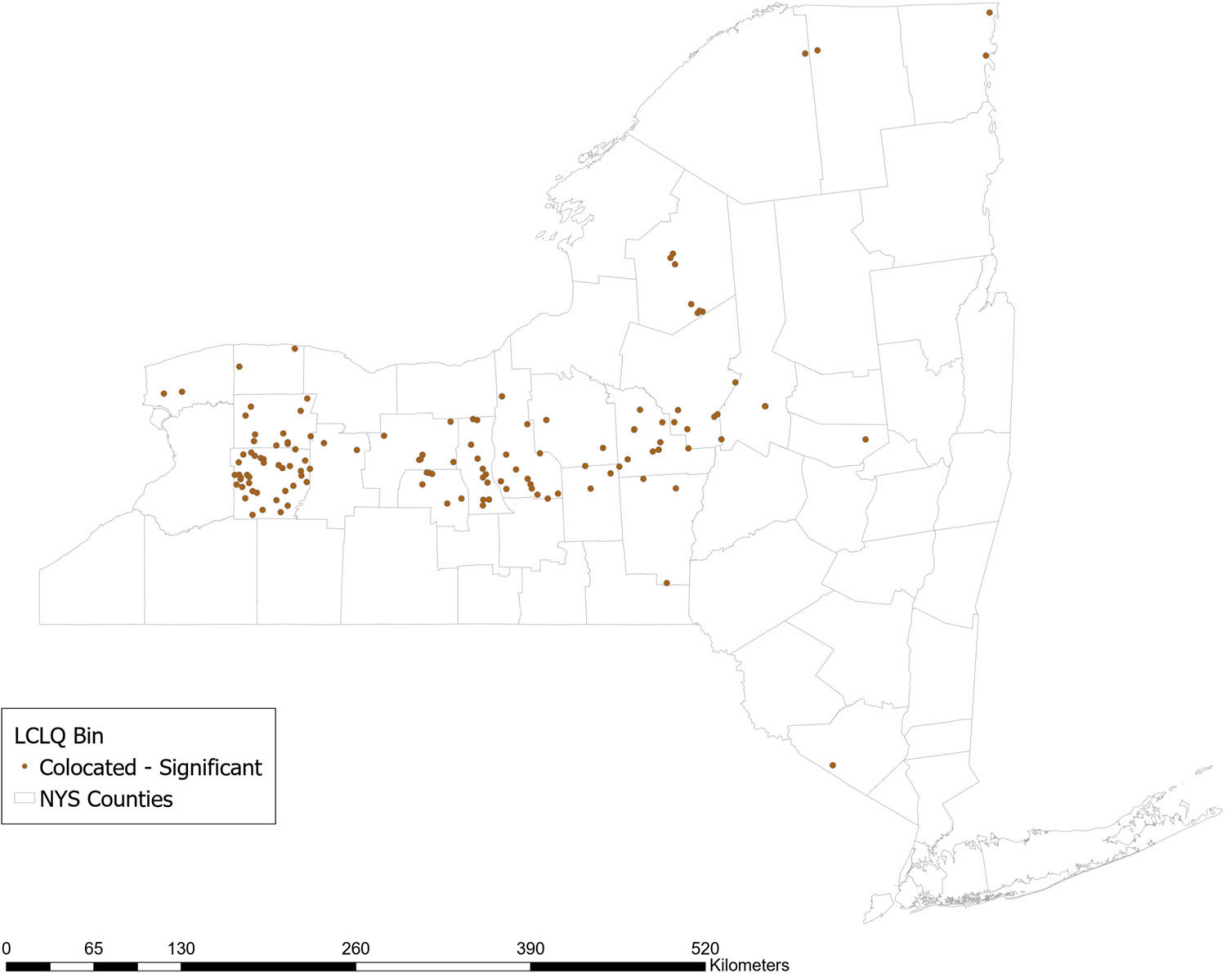
Local colocation of not using no-till methods and watershed >=1 standard deviation above mean incremental N yield from agricultural sources

Approximately 36% of respondent farms use no-till methods. The GCLQ between not using no-till methods and having a high N yield from agriculture is 2.54 (*p* = 0.02), indicating significant colocation between farms that do not use planted buffer zones and waterways with a high N yield from agriculture. Examining the LCLQ, 13.5% of farms that do not use no-till methods are collocated with a watershed with a high N yield from agriculture. The majority of those farms (shown in dark brown in Fig. 2) are located in a stretch across the center of NY state from East to West. No locations have LCLQs that indicate significant isolation.

#### Use of Soil Testing for Nutrient Content

**Fig. 3:**
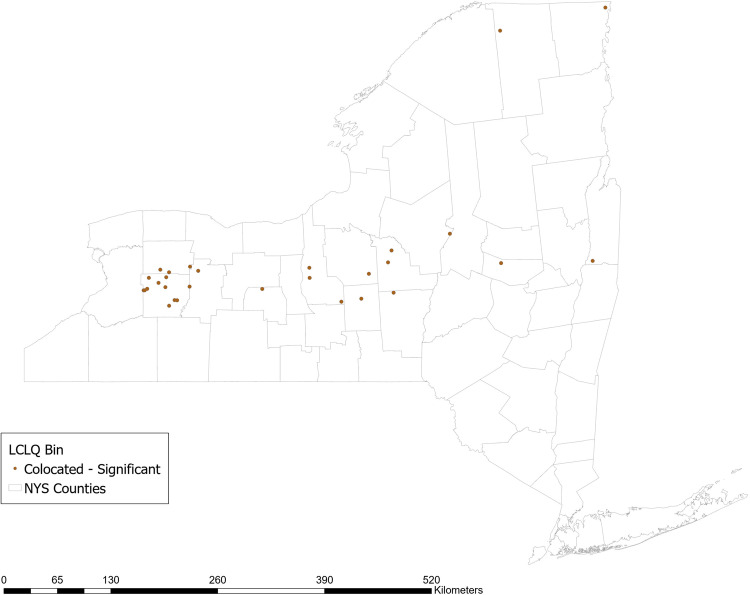
Local colocation of not using soil testing and watershed >=1 standard deviation above mean incremental N yield from agricultural sources

Approximately 75% of respondent farms report ever testing their soil for nutrient content. The GCLQ between not using soil testing and having a high N yield from agriculture is 1.71 (*p* = 0.02), indicating significant colocation between farms that do not use soil testing and waterways with a high N yield from agriculture. Examining the LCLQ, 8% of farms that do not use soil testing are collocated with a watershed with a high N yield from agriculture. The majority of those farms (shown in dark brown in Fig. 3) are located in a stretch across the center of NY state from East to West. No locations have LCLQs that indicate significant isolation.

#### Use of Planted Buffer Zones Around Waterways

**Fig. 4:**
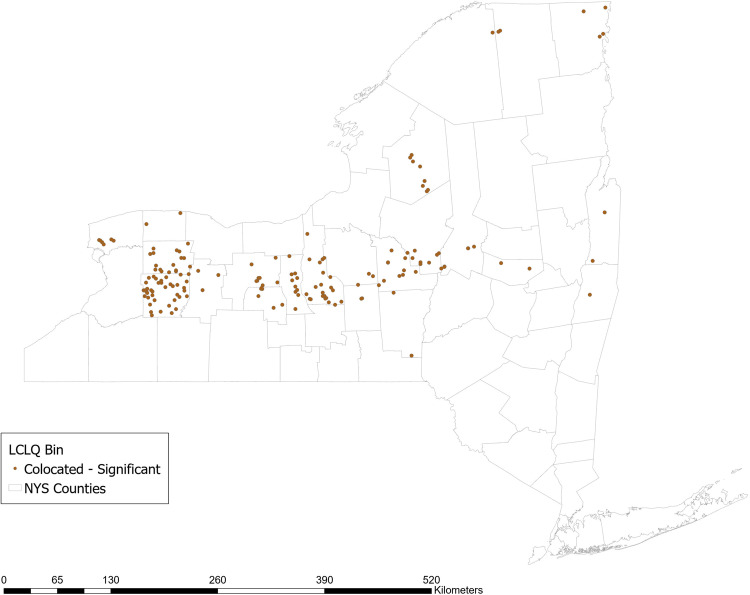
Local colocation of not using planted buffer zones and watershed >=1 standard deviation above mean incremental N yield from agricultural sources

Approximately 20% of farms that border surface waters report using planted buffer zones around waterways. The GCLQ between not using planted buffer zones and having a high N yield from agriculture is 2.54 (*p* = 0.02), indicating significant colocation between farms that do not use planted buffer zones and waterways with a high N yield from agriculture. Examining the LCLQ, 13% of farms that do not use planted buffer zones are collocated with a watershed with a high N yield from agriculture. Many of those farms (shown in dark brown in Fig. 4) are located in a stretch across the center of NY state from East to West. No locations have LCLQs that indicate significant isolation.

### Colocation of High Nutrient Yields and BMPs: Phosphorous

#### Use of No-Till Methods

**Fig. 5:**
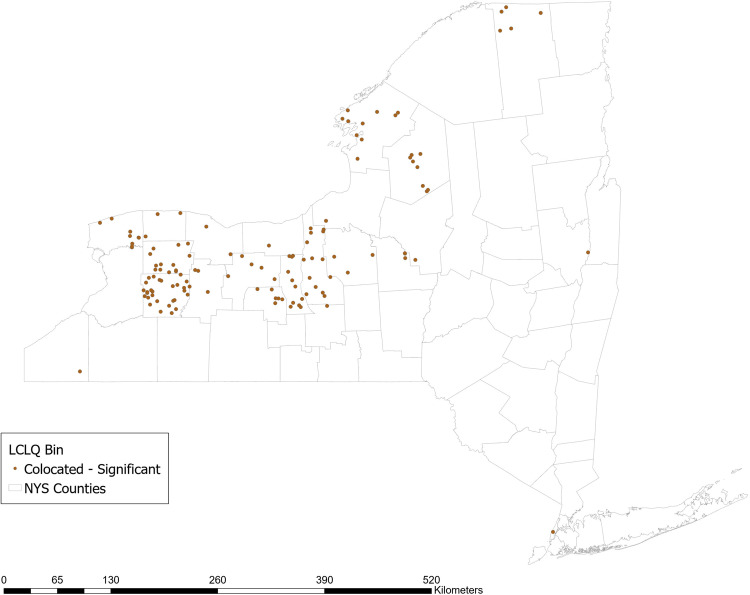
Local colocation of not using no-till methods and watershed >=1 standard deviation above mean incremental P yield from agricultural sources

The GCLQ between not using no-till methods and having a high P yield from agriculture is 2.09 (*p* value 0.02), indicating significant colocation between farms that do not use planted buffer zones and waterways with a high P yield from agriculture. Examining the LCLQ, 14% of farms that do not use no-till methods are collocated with a watershed with a high P yield from agriculture. The majority of those farms (shown in dark brown in Fig. 5) are located in Central and Western NY state. No locations have LCLQs that indicate significant isolation.

#### Use of Soil Testing for Nutrient Content

**Fig. 6:**
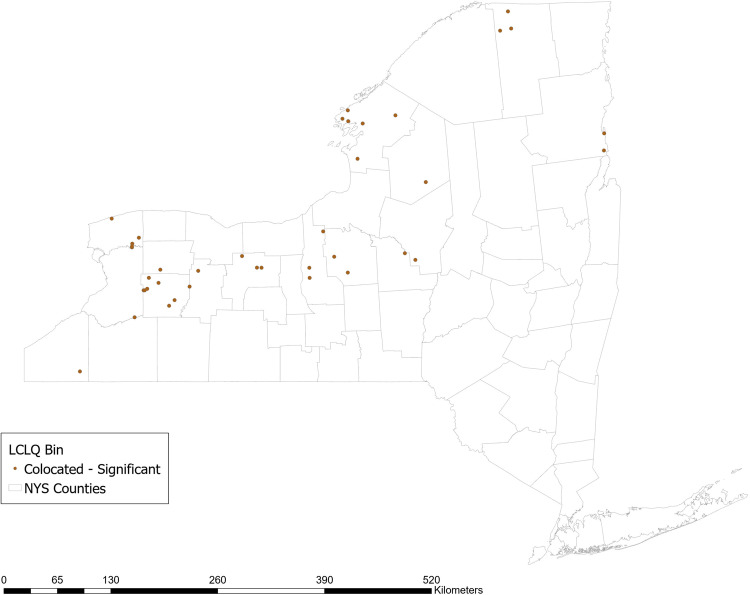
Local colocation of not using soil testing and watershed >=1 standard deviation above mean incremental P yield from agricultural sources

The GCLQ between not using soil testing and having a high P yield from agriculture is 1.66 (*p* = 0.02), indicating significant colocation between farms that do not use soil testing and waterways with a high P yield from agriculture. Examining the LCLQ, 10% of farms that do not use soil testing are collocated with a watershed with a high P yield from agriculture. Most of those farms (shown in dark brown in Fig. 6) are located in a stretch across the center of NY state from East to West. No locations have LCLQs that indicate significant isolation.

#### Use of Planted Buffer Zones Around Waterways

**Fig. 7:**
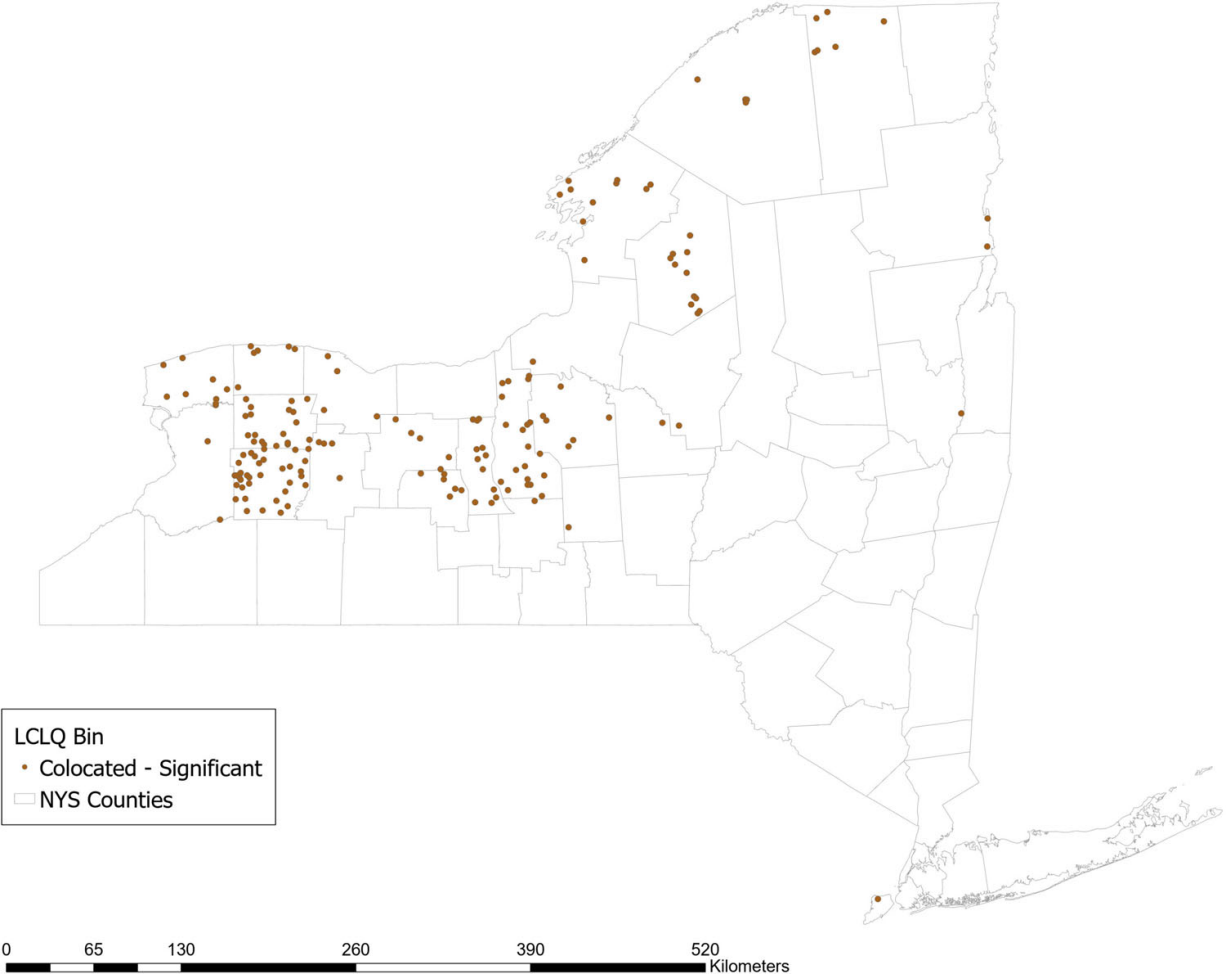
Local colocation of not using planted buffer zones and watershed >=1 standard deviation above mean incremental P yield from agricultural sources

Approximately 20% of farms that border surface waters report using planted buffer zones around waterways. The GCLQ between not using planted buffer zones and having a high P yield from agriculture is 2.05 (*p* = 0.02), indicating significant colocation between farms that do not use planted buffer zones and waterways with a high P yield from agriculture. Examining the LCLQ, 14% of farms that do not use planted buffer zones are collocated with a watershed with a high P yield from agriculture. Farms (shown in dark brown in Fig. 7) are mainly located in Central and Western NYS. No locations have LCLQs that indicate significant isolation.

## Discussion

As indicated by our representative survey, the largest commodities within NYS are hay, beef, dairy, and corn. Across these commodities, there was a strong adoption of conservation practices. As expected, most agricultural properties border surface water bodies of some kind, emphasizing the importance of conservation practices that are relevant to nutrient transport and water quality. However, no BMP is universally adopted and rates of use are lowest for the use of planted buffer zones around surface waters, a practice shown to have a significant impact on nutrient transfer (Mayer et al. 2007; Zak et al. 2018; Walton et al. 2020).

SPARROW models for NYS show some areas of the state with high incremental N and P yields from agriculture, particularly in the Central and Western portions of the state (see Appendix). Anselin Local Moran’s I also shows the clustering of watersheds with elevated levels of incremental N and P yields from agriculture in Central and Western NYS (see Fig. 1).

We find mixed support for our hypothesis that areas with low usage of BMPs would be collocated with areas having high N and P yields in waterways. Overall, all three BMPs showed a significant global collocation between not using the BMP and having an incremental N or P yield from agriculture that is more than one standard deviation above the state mean. However, no BMP showed a local collocation percentage in which more than 15% of non-adopters were collocated with a watershed with an elevated N or P yield. Taken together, this shows that there are some areas of NYS in which collocation of non-adoption and elevated N and P yields from agriculture are a concern but others in which there is no clear collocation relationship. This variation across NYS confirms our second hypothesis that the spatial relationship between BMPs and N and P yields in waterways will vary spatially, reflecting local biogeophysical and social conditions. The spatial variation in BMP adoption, N and P yields, and the relationship between the two confirms the need for further studies of spatial patterns of conservation behaviors and water quality.

Our colocation analysis was designed to identify potential areas of concern that could be targeted for future monitoring and/or intervention by delineating areas in which high incremental N or P yields from agriculture are collocated with the non-use of BMPs. Identifying these priority areas could allow state agencies and conservation partners to target their limited resources for outreach and extension. The colocation analysis identifies several areas of Central and Western NYS where targeted intervention may be particularly helpful. Particularly regarding the use of planted buffer zones around surface waters, there is a strong and statistically significant colocation of elevated incremental yields of both N and P and a very low rate of buffer zone adoption in the areas. The same areas also show strong and significant colocation of elevated incremental yields of both N and P and a very low rate of adoption of no-till methods. These findings suggest that outreach and extension efforts should be targeted in these regions of Central and Western NYS including the so-called Finger Lakes which are very water-dense. State agencies may have potential partners in the area to support outreach and extension, including several very active lake associations that are focused on local water quality issues. Lake associations are active throughout the Finger Lakes and already promote soil testing and other BMPs to farmers in the region (Cayuga Lake Watershed Network 2021). Previous research in Western NYS demonstrated the effectiveness of BMPs promoted by local watershed and lake associations to reduce runoff into Conesus Lake, particularly soil testing (Herendeen and Glazier 2009). These regional groups and state partners should target outreach and promotion of BMP adoption in the regions of the state that have both high incremental N and P yields from agriculture and non-adoption of our focal BMPs.

The observed colocation between modeled yields of nutrients and areas of high (or low) adoption of BMP has several additional implications for nutrient and water quality management. First, it is encouraging that we see many conservation practices clustered in areas with N and P yields greater than the statewide mean. This indicates that the practices necessary to address surface water quality issues are being adopted within the areas where such practices are most crucial. This approach also emphasizes the importance of investigating multiple conservation practices in tandem. As we show, there is a strong regional preference for the use (and avoidance) of certain conservation practices. This extends the work by Zimmerman and colleagues (Zimmerman et al. 2019b) that finds that farmers are aware of spatial variation in the appropriateness of conservation practices in the US Midwest. While our analysis can show general colocation between patterns of modeled water quality and reports of conservation behavior adoption, a mechanistic model analysis would be required to determine whether these conservation practices are leading directly to reductions in levels of N and P. Many studies have also demonstrated that we can anticipate lags between the application of manure/fertilizer to the land and nutrient load response (Meals et al. 2010; Van Meter et al. 2017), such that it can be years before we see any water quality benefits from best management practices, especially within large watersheds. Our findings do suggest, however, the need for further spatial analysis of BMP adoption and water quality measures and longitudinal data collection that could better address causal mechanisms.

The overall impact of agriculture on water quality is well understood (Sharpley and Jarvie 2012) and the use of conservation tillage (Yang et al. 2005; Logan et al. 2018; Kelly et al. 2019; Cooper et al. 2020), regular soil testing for nutrient content (Gartley and Sims 1994a; Sims 1998a; Maguire and Sims 2002), and buffer zones or conservation cover around waterways (Norris 1993; Lovell and Sullivan 2006) have been demonstrated to influence nutrient runoff to waterways. Moreover, we know that conservation behaviors interact with the features of the local watersheds and waterways to influence their effectiveness (Yang et al. 2005; Gurdak and Qi 2012; Zimmerman et al. 2019a; Cheng et al. 2020). What our spatial analyses offer is the ability to empirically identify areas in which non-adoption of conservation behaviors is collocated with high incremental N and P yields from agriculture across a state that is incredibly water-dense. Together, our analyses highlight several areas within the state that would be suitable for long-term water quality monitoring to ascertain whether hot spots of BMPs are having the intended impact on in-stream water quality.

### Limitations and Considerations

Our analysis relies on measures of N and P yields from agriculture generated from SPARROW models (Schwarz et al. 2006). While SPARROW models have been demonstrated to provide valid and reliable estimates of contaminants and their sources (Preston et al. 2011; Saad et al. 2011), they are not direct observations of nutrient load/yield in waterways. In an ideal case, we would compare observations of BMP adoption to variations in nutrient yields from surface water bodies through time. This is not possible with SPARROW due to its empirical mass-balance approach. Using more complex physically-based mechanistic nutrient load models, such as SWAT, WASP, or AnnAGNPS among others, is another approach that can determine nutrient load variations through time and space, though it is mostly applicable to watersheds across the US where in-situ water quality measurements are available for model calibration and verification (Merriman et al. 2018; Wool et al. 2020; Tamanna et al. 2020).

Similarly, our survey data on BMPs was collected at a single point in time. We are therefore unable to assess how conservation behaviors may have varied over time. There is a variable lag time between the adoption of BMPs and nutrient yield improvements, particularly P, (Meals et al. 2010) that we are unable to address. Finally, BMPs are reported by respondents and geolocated to the primary address of the farm, which may be different than the mailing address of the farm owner. However, this approach was chosen given the large sizes of farms (the average farm size is approximately 199 acres) and the lack of information regarding the specific spatial location of where each BMP is practiced on the farm. While we anticipate there could be some variation in statistical results if the specific location of each BMP were codified, we anticipate the distances between farms are much greater than the uncertainty in BMP location within each farm.

## Conclusion

Excess N and P in waterways is associated with a wide range of environmental and health risks, including biodiversity loss (Smith et al. 1999; Dodds and Smith 2016; Guignard et al. 2017), toxic algal blooms (Michalak et al. 2013), and millions of lost revenue (Dodds et al. 2009). Agricultural application of manure and fertilizer is the primary cause of excess nutrient loads in waterways (Laitos and Ruckriegle 2012; Longhurst 2012; Sharpley and Jarvie 2012; Centner 2012) and agricultural BMPs have been promoted to reduce nutrient runoff and improve water quality. Our findings show that the adoption of these three BMPs varies spatially and that non-adoption of BMPs may collocate with high N and P yields in some areas. This spatial understanding of BMP usage and nutrient yields can inform outreach and decision-making in regions where BMP adoption could be improved and identify priority areas to target.

The use of spatial analysis is critical to future understanding and policy regarding BMPs and nutrient inputs to waterways. Our analysis combines measures of nutrient yield with reported use of agricultural BMPs, allowing us to examine spatial colocation between BMPs and nutrient yields. These methods are generalizable to other settings and contexts to develop a more complex and localized understanding of how BMPs interact with diverse biogeophysical and social contexts, and longitudinal datasets on both water quality and conservation behaviors would allow for more specific causal modeling. We encourage extending the spatial examination of BMPs and nutrient yields across a range of diverse watersheds and regions (Yang et al. 2005; Gurdak and Qi 2012; Zimmerman et al. 2019a; Cheng et al. 2020) to identify the most effective and spatially targeted BMPs for their region. By understanding the spatial context of BMP adoption, we can better target areas for water quality monitoring and further outreach to benefit surface water quality issues now and into the future.

## Supplementary information

Supplementary Information

Survey

Appendix

## Data Availability

Data not including identifying information is available upon request.
