# Supplementary material for: Spatial Variation in Agricultural BMPs and Relationships with Nutrient Yields Across New York State Watersheds
Source: Environ Manage. 2024 Jul 2;74(4):729–41. doi: 10.1007/s00267-024-02008-x (PMC11392999; doi:10.1007/s00267-024-02008-x)
Supplement: Supplementary file 1 — Supplementary Information [file 267_2024_2008_MOESM1_ESM.docx]

**SUPPLEMENTARY INFORMATION**


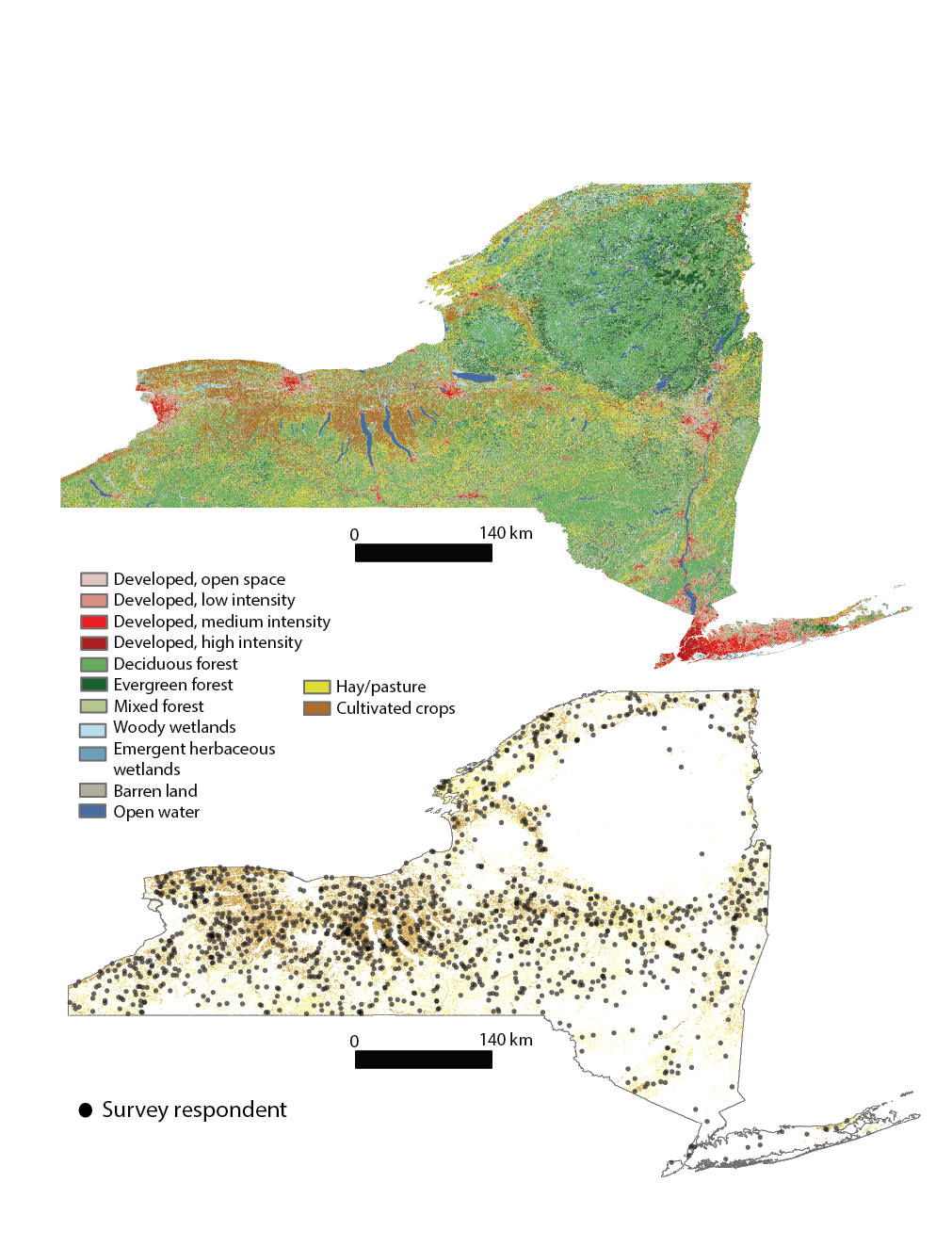


Figure S1: Land cover information for 2019 (top) and survey respondents (bottom) across NYS. Survey respondents are shown against a backdrop of agricultural land cover (hay/pasture and cultivated crops (Multi-Resolution Land Characteristics consortium 2019) .

Table S1: Mail Survey Sample

|  |  | NY Farm Population* (farms) | Sampled Farms | Usable Survey Responses |
| --- | --- | --- | --- | --- |
| Hay | <100 acres | 7,420 | 1716 | 283 |
|  | 100+ acres | 117 | 117 | 38 |
| Beef | <100 head | 1,784 | 908 | 303 |
|  | 100+ head | 56 | 50 | 14 |
| Dairy | <100 head | 4,879 | 880 | 216 |
|  | 100+ head | 2,274 | 760 | 187 |
| Corn | <100 acres | 12,208 | 1225 | 249 |
|  | 100+ acres | 527 | 527 | 184 |
|  |  |  | 6,183 | 1,474 |

* 2017 Census of Agriculture (USDA National Agricultural Statistics Service 2017)

Table S2: One Sample T-test of Sample Representativeness

|  | Farm Size |
| --- | --- |
| Sample mean | 199 acres |
| Census mean* | 205 acres |
| Standard error | 10.9192 |
| n | 1474 |
| t | 0.8242 |
| Two-tailed p value | 0.4099 |

* Mean for NY state from the 2017 Census of Agriculture (USDA National Agricultural Statistics Service 2017)


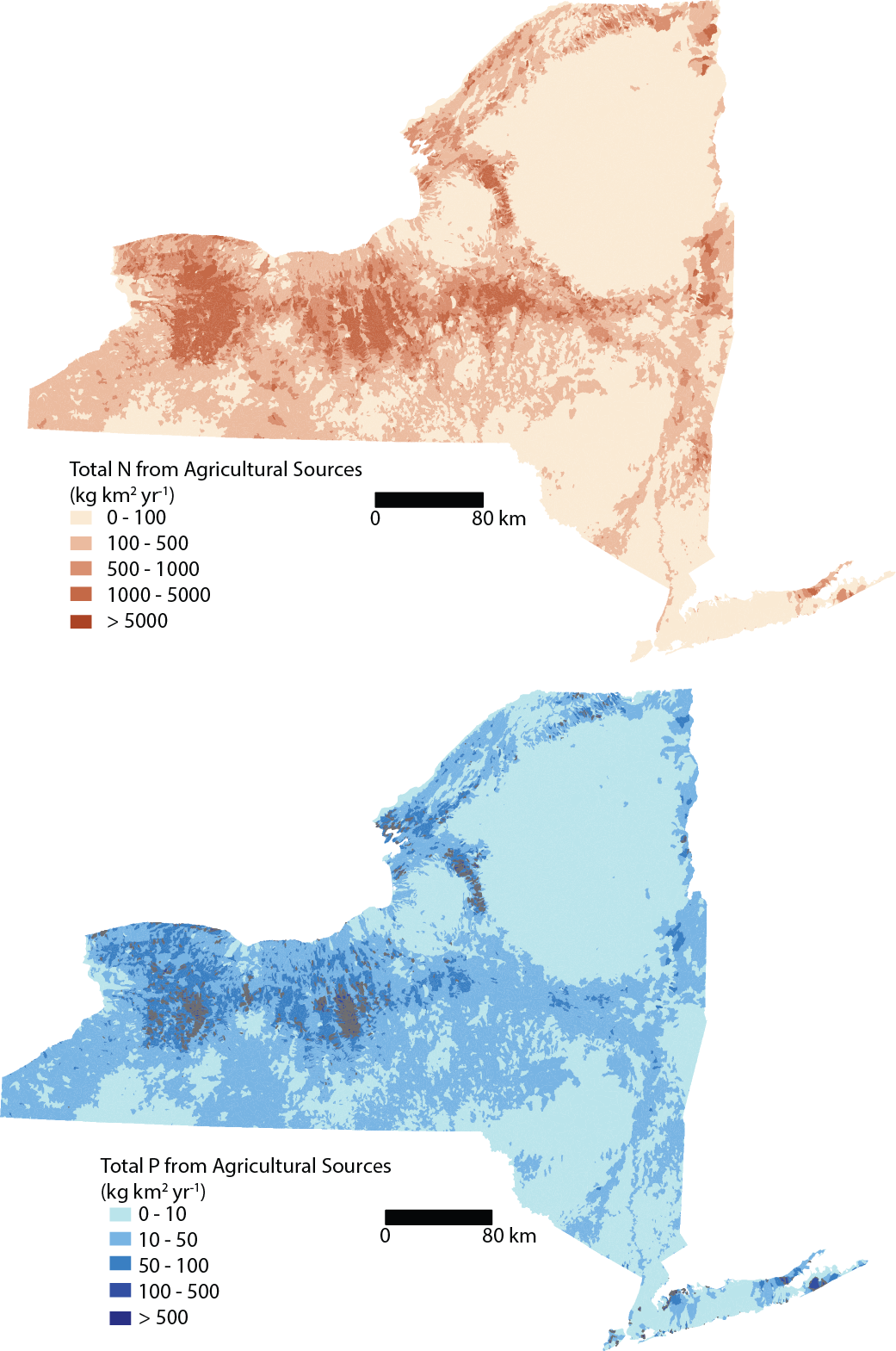


Figure S3: Total N (top) and P (bottom) derived from agricultural sources, as estimated by the USGS SPARROW models for Northeast and Midwest regions (Ator 2019; Robertson and Saad 2019).
