# Supplementary material for: Spatial Variation in Agricultural BMPs and Relationships with Nutrient Yields Across New York State Watersheds
Source: Environ Manage. 2024 Jul 2;74(4):729–41. doi: 10.1007/s00267-024-02008-x (PMC11392999; doi:10.1007/s00267-024-02008-x)
Supplement: Supplementary file 2 — Survey [file 267_2024_2008_MOESM2_ESM.pdf]

Syracuse University

Manure and Biosolids Agricultural Survey

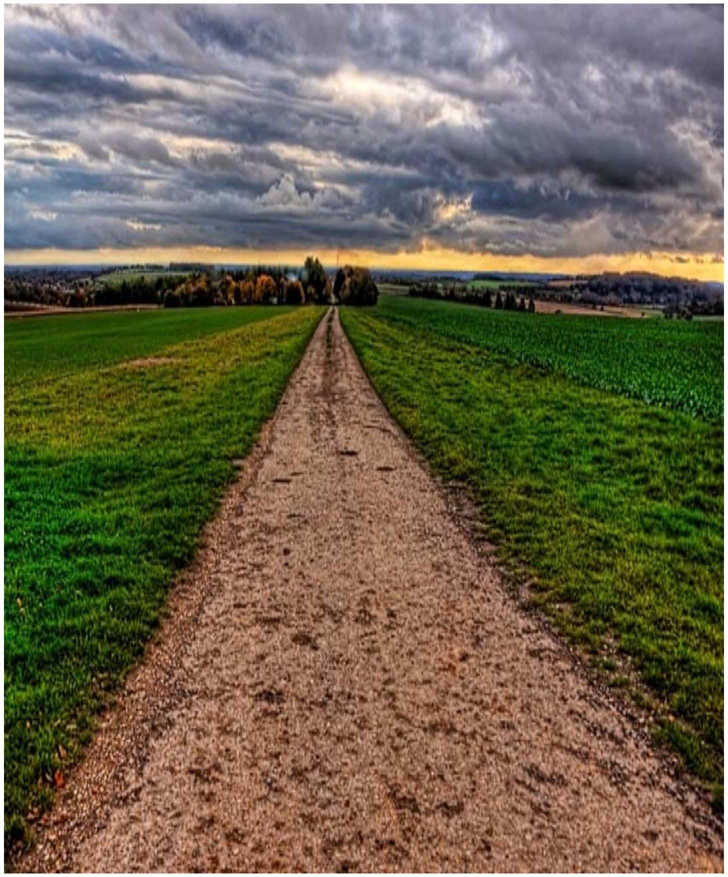

First, we would like to know about your farm.

1. **Have you engaged in agricultural production of any scale within the last two years?**
   - ○ Yes, I am currently in agricultural production.
   - ○ Yes, I was in agricultural production within the last two years but am not currently.
   - ○ No, I have not been in agricultural production within the last two years. ***(If no, you may end the survey now.)***

2. **What agricultural products do you currently produce? Please indicate the amount. *Write 0 if none.***

**Animal Production:**	
a. Beef: Approximate beef animal units (including any cattle for meat production):	________
b. Dairy: Approximate milking cows:	________
c. Poultry: Approximate poultry animal units:	________
d. Pork/swine: Approximate swine animal units:	________
e. Other animal production:	
Type of animal:	________
Approximate animal units:	________

**Crop Production:**	
f. Corn: Approximate acres:	________
g. Soy: Approximate acres:	________
h. Wheat: Approximate acres:	________
i. Hay: Approximate acres:	________
j. Other crop production:	
Please indicate type:	________
Approximate acres:	________

3. **How much total farmland do you manage (both owned and rented, but not rented out to others)?**

   a. Acres owned: ____________________

   b. Acres rented: ____________________

4. **How much of the land you own or rent is irrigated?**

   Total irrigated acres: ____________________

Thank you for your time and effort!

To return this questionnaire, simply seal it with the white removable seal, and drop it in the mail.

(Return postage has been covered).

5. What soil types are most common on your farm? ***(Fill in all that apply)***

- O Sand
- O Silt
- O Clay
- O Loam
- O Other (please specify): ______________________
- O Don't know

6. Does any of the land you manage contain or border any surface water? ***(Fill in all that apply)***

- O No (SKIP TO QUESTION 8)
- O Yes, lake or pond
- O Yes, river or creek
- O Yes, ditch or gully
- O Yes, marsh or swamp
- O Don't know

7. IF YES TO QUESTION 6: Do you utilize any buffer zones around surface water?
Buffer zones are strips of land surrounding surface water where there is no crop planted. They provide a "buffer" between the cropland and the surface water. They may be unplanted or planted with some sort of permanent vegetation such as bushes or trees.

- O No
- O Yes, unplanted buffer zone: __________ feet
- O Yes, planted buffer zone: __________ feet
- O Don't know

8. What kind of tillage do you use? ***Please indicate all types that apply for the fall and spring seasons.***

	Fall	Spring
Chisel plow	O	O
Moldboard plow	O	O
Strip/zone/row	O	O
Disk	O	O
No till	O	O

9. How often is soil tested for nutrient content?

- O Every year
- O Every 1-5 years
- O Less than every five years
- O Never

Manure

10. **During the previous season, was animal manure (in any form) applied to land you own or rent?**
    - O Yes (please continue)
    - O No (SKIP AHEAD TO QUESTION 19)

11. **Who had primary responsibility for making manure application decisions?**
    - O Myself (owner or manager)
    - O Other owner or manager
    - O Employees (not owner or manager)
    - O Custom applicator
    - O Landowner (if you rent)
    - O Other (please specify): ______________________________

12. **What type of manure was applied? *(Fill in all that apply)***
    - O Dairy
    - O Beef
    - O Poultry
    - O Swine
    - O Other (please specify): ______________________________
    - O Don't know

13. **What form was the manure applied in?**
    - O Liquid
    - O Solid
    - O Both liquid and solid
    - O Don't know

14. **Was the manure tested for nutrient content (nitrogen content, phosphorous content, etc.)?**
    - O No
    - O Yes
    - O Don't know

15. **Did you use a calculated manure application rate?**
    - O No
    - O Yes
    - O Don't know

42. **What vear were you born?**

    |_|_|_|_|

43. **What is your gender?**
    - O Man
    - O Woman
    - O Prefer not to say

44. **What was your annual household income during 2018?**

    $ ______________________________

45. **What percentage of your household income came from agriculture in 2018?**

    ____________ %

46. **What is your highest level of education?**
    - O Some formal schooling
    - O High school degree or equivalent
    - O Some college
    - O Associates or technical degree
    - O Bachelor's degree
    - O Postgraduate degree

Biosolids Intention

38. Do you have plans to apply biosolids to land you own or rent in the future?

- O No (SKIP TO QUESTION 41)
- O Yes
- O Don't know

39. When do you plan to apply the biosolids?

- O Next season
- O Sometime within the next 2-5 years
- O Sometime within the next 5-10 years
- O Not for at least 10 years

40. Where do you plan to acquire the biosolids?

____

Water Quality

41. Below is a list of water pollutants that can become a problem when present in excessive amounts. In general, how much of a problem are the following pollutants in the area where you own or rent farmland?

	Not a problem	Slight problem	Moderate problem	Severe problem
Sediment/silt	O	O	O	O
Nitrate	O	O	O	O
Phosphorus	O	O	O	O
Bacteria in the water (such as E.coli)	O	O	O	O
Pesticide	O	O	O	O
Human pharmaceutical residue	O	O	O	O
Animal pharmaceutical residue	O	O	O	O
Household chemicals	O	O	O	O
Heavy metals	O	O	O	O

16. During the previous season, approximately how much manure was applied in each month? ***Write 0 if none was applied. Write "dk" if you don't know the approximate amount.***

Month	Total Dry Tons Applied (solid)	Total Gallons Applied (liquid)
January	________ tons	________ gallons
February	________ tons	________ gallons
March	________ tons	________ gallons
April	________ tons	________ gallons
May	________ tons	________ gallons
June	________ tons	________ gallons
July	________ tons	________ gallons
August	________ tons	________ gallons
September	________ tons	________ gallons
October	________ tons	________ gallons
November	________ tons	________ gallons
December	________ tons	________ gallons

17. During the previous season, would you say that the amount of manure applied was typical for a season?

- O Yes, applied an amount simliar to most seasons
- O No, applied more than most seasons
- O No, applied less than most seasons
- O Don't know

18. How important are each of the following sources of information when making MANURE application decisions?

	Not at all important	Slightly important	Somewhat important	Very important
Weather	O	O	O	O
Maximum yield potential	O	O	O	O
Past experience/history	O	O	O	O
Recommended rate from soil test	O	O	O	O
Advice from fertilizer dealer	O	O	O	O
Advice from Cornell Cooperative Extension	O	O	O	O
Advice from magazines or journals	O	O	O	O

Biosolids

Biosolids are nutrient-dense organic materials produced from municipal waste that is processed at a wastewater treatment plant. Biosolids are <u>not the same as septage</u>, which is a raw, untreated waste product directly removed from septic tanks and other sources. Biosolids can be applied for agricultural or other land-use.

For the questions below, biosolids can include either materials purchased directly from a wastewater treatment plant or Class A materials purchased as pelletized or bagged biosolids material.

19. Have biosolids (organic matter recycled from treated wastewater) ever been applied to land you own or rent?

- O Yes (please continue)
- O No (SKIP AHEAD TO QUESTION 31)

20. What was the most recent year that biosolids were applied to land you own or rent? ____

34. I would support the land application of biosolids for agriculture in New York State.

- O Strongly disagree
- O Somewhat disagree
- O Neither disagree nor agree
- O Somewhat agree
- O Strongly agree

35. I would support the land application of biosolids for agriculture in my county.

- O Strongly disagree
- O Somewhat disagree
- O Neither disagree nor agree
- O Somewhat agree
- O Strongly agree

36. I would support the land application of biosolids for agriculture by my neighbors.

- O Strongly disagree
- O Somewhat disagree
- O Neither disagree nor agree
- O Somewhat agree
- O Strongly agree

37. I would support the land application of biosolids for agriculture on my farm.

- O Strongly disagree
- O Somewhat disagree
- O Neither disagree nor agree
- O Somewhat agree
- O Strongly agree

Biosolids Information

Biosolids are nutrient-dense organic materials produced from municipal waste that is processed at a wastewater treatment plant. Biosolids can be applied for agricultural or other land-use.

31. How familiar are you with the use of biosolids in agricultural production?

- O Not at all familiar, have never heard of biosolids use prior to now
- O Unfamiliar, have heard of biosolids use but have limited knowledge of their use
- O Moderately familiar, know of biosolids and their use in general
- O Very familiar, know of biosolids and their use in specific cases or contexts

32. What would you describe as possible benefits of biosolids for use in agricultural production? *(Fill in all that apply)*

- O Improving soil fertility
- O Reducinig landfill waste
- O Reducing incinterator use
- O Other (please specify): ____________________
- O Don't know

33. What would you describe as possible concerns of biosolids for use in agricultural production? *(Fill in all that apply)*

- O Runoff into surface or groundwater
- O Transfer of pharmaceutical residue
- O Transfer of heavy metals
- O Transfer of household chemicals
- O Bacteria
- O Other (please specify): ____________________
- O Don't know

21. If you have stopped application of biosolids to land you own or rent, what was the primary reason for discontinuing application?

- O No applicable (still applying biosolids)
- O Cost
- O Availability
- O Municipal ban
- O Other (please specify): ____________________

22. Who had primary responsibility for making biosolids application decisions?

- O Myself (owner or manager)
- O Other owner or manager
- O Employees (not owner or manager)
- O Custom applicator
- O Landowner (if you rent)
- O Other (please specify): ____________________

23. Where did you acquire the biosolids? *(Fill in all that apply)*

- O Purchased from county or municipality.
  Please list county or municipality: ____________________
- O Purchased from waste water treatment facility.
  Please list facility: ____________________
- O Purchased from nutrient supplier
- O Other (please specify): ____________________

24. What was the primary form that the biosolids were applied in?

- O Pellets
- O Liquid
- O Dry solids
- O Compost
- O Both liquid and dry solids
- O Other (please specify): ____________________
- O Don't know

25. Were the biosolids tested for nutrient content (nitrogen content, phosphorous content, etc.)?

- O No
- O Yes
- O Don't know

26. What was your primary reason for applying biosolids?

- O Fertilizer, nutrient input
- O Waste disposal
- O Other (please specify): ___________________________________

27. If biosolids was applied to your land during <u>the previous season</u>, approximately how many dry tons of biosolids was applied in each month? ***<u>Write 0 if none was applied. Write "dk" if you don't know the approximate amount.</u>***

Month	Total Dry Tons Applied
January	__________ tons
February	__________ tons
March	__________ tons
April	__________ tons
May	__________ tons
June	__________ tons
July	__________ tons
August	__________ tons
September	__________ tons
October	__________ tons
November	__________ tons
December	__________ tons

28. If biosolids was applied to your land during <u>the previous season</u>, would you say that the amount applied was typical for a season?

- O Yes, applied an amount simliar to most seasons
- O No, applied more than most seasons
- O No, applied less than most seasons
- O Don't know

29. Have you ever held a New York State Department of Environmental Conservation permit related to biosolids?

- O No, never held permit
- O Yes, hold current permit
- O Yes, held permit previously (but not currently)

<u>Sources of Information</u>

30. How important are each of the following sources of information when making BIOSOLIDS application decisions?

	Not at all important	Slightly important	Somewhat important	Very important
Weather	O	O	O	O
Maximum yield potential	O	O	O	O
Past experience/history	O	O	O	O
Recommended rate from soil test	O	O	O	O
Advice from fertilizer dealer	O	O	O	O
Advice from Cornell Cooperative Extension	O	O	O	O
Advice from magazines or journals	O	O	O	O
