# Supplementary figures and images for: Spatial Variation in Agricultural BMPs and Relationships with Nutrient Yields Across New York State Watersheds
Source: Environ Manage. 2024 Jul 2;74(4):729–41. doi: 10.1007/s00267-024-02008-x (PMC11392999; doi:10.1007/s00267-024-02008-x)

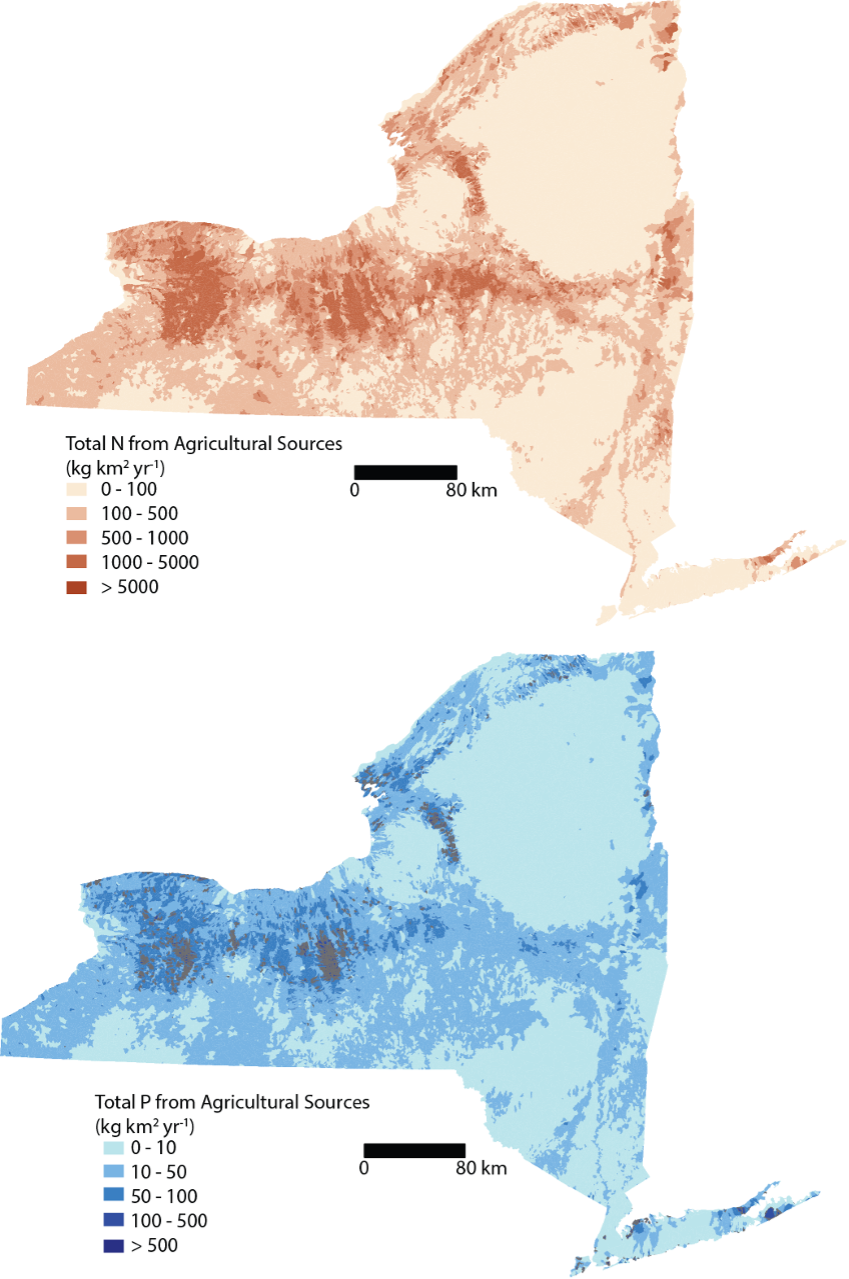

Supplement: Supplementary file 3 — Appendix [file 267_2024_2008_MOESM3_ESM.png]
